# Bachmann’s Bundle Is Derived From Odd Skipped Related-1–Expressing Myocardial Progenitors and Facilitates Interatrial Conduction in the Murine Heart

**DOI:** 10.1016/j.jacep.2026.02.012

**Published:** 2026-03-19

**Authors:** Giselle Galang, Gagandeep Chouhan, Amanda Soe, Joseph Bayne, Ravi Mandla, Di Lang, Vasanth Vedantham

**Affiliations:** Cardiology Division, Department of Medicine and Cardiovascular Research Institute, University of California-San Francisco, San Francisco, California, USA.

**Keywords:** Bachmann’s bundle, cardiac conduction system, cardiac development, interatrial conduction, Osr1

Bachmann’s bundle (BB) is a band of retroaortic interatrial myocardial fibers responsible for impulse transmission from the sinoatrial node to the left atrium.^1^ Failure of conduction along BB causes interatrial conduction delay and an increased risk of atrial fibrillation (AF), whereas BB pacing may reduce the incidence of AF.^2^ The embryological origins of BB have not been defined, nor has interatrial conduction via BB been explored in a genetically tractable animal model.

All animal experiments were performed in accordance with an approved Institutional Animal Care and Use Committee protocol at the University of California-San Francisco. Whole mount light microscopy of an adult mouse heart revealed a discrete myocardial band at the expected location of BB (Figures 1A and 1B). Using optical mapping and right atrial pacing of an ex vivo perfused atrial preparation configured to not disrupt interatrial connections, we observed robust right-to-left atrial conduction with an average conduction velocity of 46 ± 2 cm/second (Figure 1C). BB was then severed in 4 of 6 hearts; in the remaining 2, only the interatrial septum was cut, sparing BB. There was no difference in conduction velocity in these 2 groups before the cuts: 46 ± 2 cm/second before cutting BB (n = 4) vs 45 ± 2 cm/second before the septum cut (n = 2). A delay in left atrial activation occurred in all BB cuts as visualized by isochronal activation maps and by the timing of activation at a specific fiducial point in the left atrium (Figure 1D).

A subtractive map of atrial activation delay due to the BB cut showed delay only distal to the incision in the left atrium but not in the right atrium (Figure 1E), with significant slowing of conduction velocity in all 4 of the full thickness cuts (34 ± 2 cm/second; n = 4) but in neither of the partial cuts (43 ± 3 cm/second; n = 2, not shown) (Figure 1F). By recapitulating the findings of Bachmann from canine hearts,^1^ these data show the deep mammalian conservation of BB structure and function.

The dorsal mesenchymal protrusion (DMP),^3^ a retrocardiac mesenchymal progenitor population that forms the left venous valve and interatrial septum, transiently expresses the transcription factor Odd-skipped related-1 (*Osr1*).^4^ *OSR1*^*GCE*^ knock-in/knockout mice (JAX strain #009061; The Jackson Laboratory) express green fluorescent protein fused to tamoxifen-inducible Cre recombinase from the endogenous *Osr1* genomic locus, allowing visualization and fate mapping of the DMP.^5^ Expression of the *GFP-CreERT2* allele in embryonic day 11.5 *Osr1*^*GCE/*+^ hearts (Figure 1G) recapitulated the expression pattern of *Osr1* messenger ribonucleic acid in the DMP (Figure 1H). To visualize DMP fate, we crossed *Osr1*^*GCE/*+^ with *ROSA*^*LacZ/LacZ*^ mice (JAX Strain #002073; The Jackson Laboratory), induced Cre recombinase with tamoxifen at embryonic day 10.5, collected embryos at embryonic day 14.5, and stained them with 5-bromo-4-chloro-3-indolyl-β-D-galactopyranoside. As expected, the interatrial septum and left venous valve exhibited robust reporter activity (Figure 1I). We also observed a contiguous band of labeled myocardium extending from the perinodal right atrium across the right atrial–left atrial junction and terminating at the trabeculated portion of the left atrium, in the expected location of BB, thereby establishing BB as a DMP derivative (Figures 1I and 1J).

Homozygous deletion of Osr1 causes embryonically lethal cardiac morphogenetic defects.^4^ *Osr1*^*GCE/*+^ mice are haploinsufficient for Osr1, raising the possibility that cardiac conduction could be affected in these mice. A 48-hour electrocardiogram recording using implanted transmitters (ETA-F10; Data Science International) revealed no difference in mean heart rate averaged over 48 hours of continuous recording between *Osr1*^*GCE/*+^ and wild-type littermates, nor any difference in P-wave duration, reflecting grossly intact impulse generation and interatrial conduction despite Osr1 haploinsufficiency (Figure 1K).

Taken together, these data establish the mouse as a genetically tractable model system for exploration of interatrial conduction via BB. *Osr1*^*GCE*^ mice can be used for selective genetic modification of DMP-derived structures, including BB, potentially facilitating molecular exploration of the pathways downstream of interatrial conduction delay.

## Figures and Tables

**FIGURE 1:**
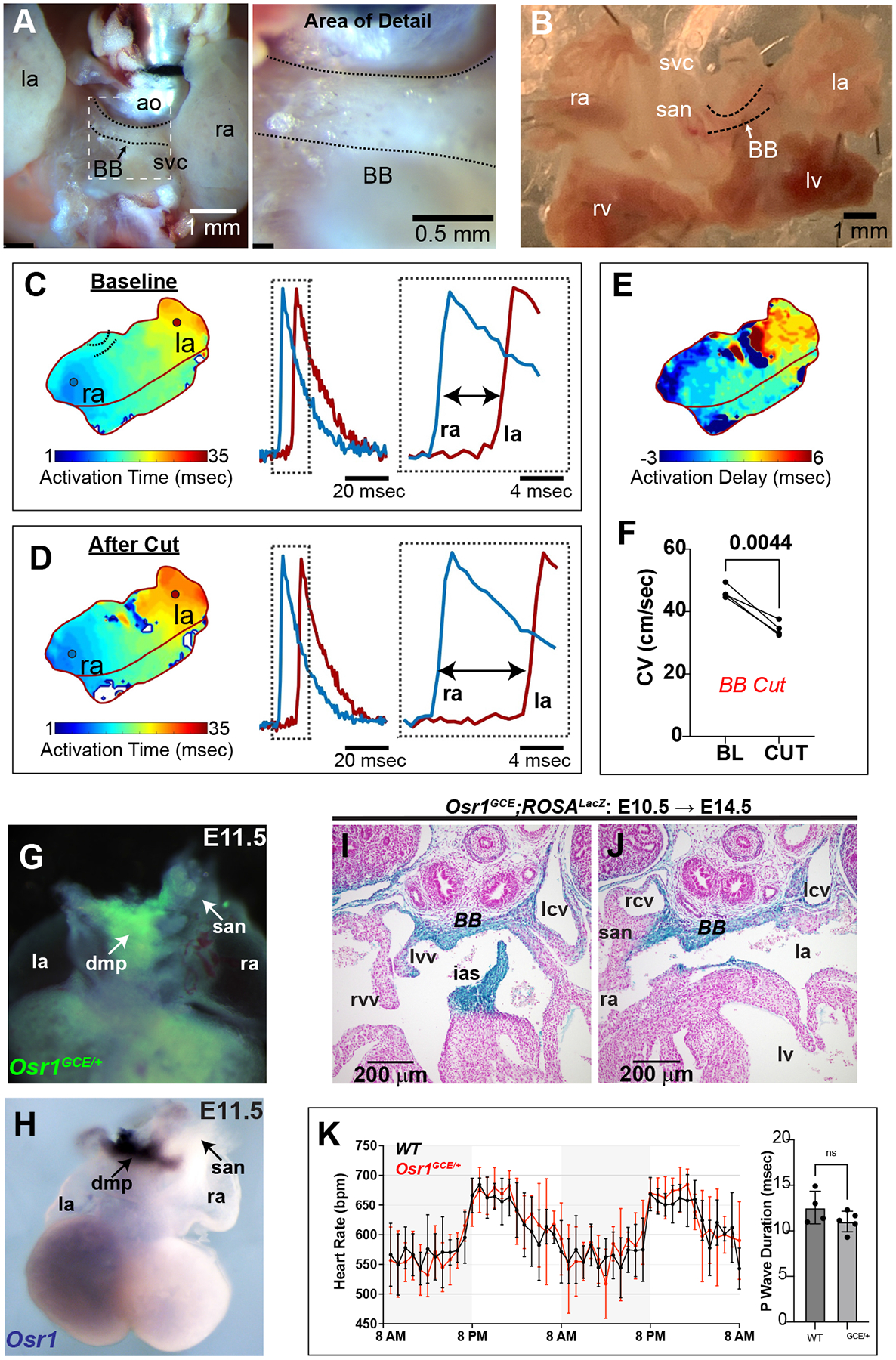
Functional and Embryological Characterization of Murine Bachmann’s Bundle (A) Adult wild-type (WT) mouse heart showing Bachmann’s bundle (BB) (dashed lines), an interatrial bundle spanning the region between the superior vena cava (SVC) and the aorta (ao) (area of detail magnified at right). (B) Ex vivo atrial preparation with the endocardial aspect of BB indicated. Isochronal map of an ex vivo atrial preparation before (C) and after (D) severing BB, alongside optical activation tracings from right atrial (blue) and left atrial (red) fiduciary points. (E) Map of activation delay at each atrial point caused by severing BB. (F) Change in conduction velocity (CV) between baseline (BL) and after severing BB (n = 4). (G) Whole mount *Osr1*^*GCE/*+^ embryonic day 11.5 (E11.5) heart displays specific green fluorescent protein expression in dorsal mesenchymal protrusion (dmp). (H) In situ hybridization for *Osr1* messenger ribonucleic acid at E11.5. (I and J) *Osr1*^*GCE/*+^ *; ROSA*^*LacZ/*+^ E14.5 heart sections after induction with tamoxifen at embryonic day 10.5 (E10.5), staining with 5-bromo-4-chloro-3-indolyl-β-D-galactopyranoside, sectioning and counterstaining with Nuclear Fast Red shows the fate of dmp in the interatrial septum (ias), left venous valve (lvv), and BB. (K) Comparison of hourly heart rate averages over 48 hours in awake and unrestrained WT mice (n = 4) and *Osr*^*GCE/*+^ adult mice (n = 4) alongside a comparison of P-wave duration. Comparisons were made with a Student’s *t*-test, and error bars denote SDs. E14.5 = embryonic day 14.5; la = left atrium; lcv = left cardinal vein; lv = left ventricle; ns = not significant; ra = right atrium; rcv = right cardinal vein; rv = right ventricle; rvv = right venous valve; san = sinoatrial node.
